# Mexiletina em um Recém-Nascido com Síndrome do QT Longo Tipo 3: Quando o Acesso se Impõe à Urgência

**DOI:** 10.36660/abc.20210533

**Published:** 2022-05-04

**Authors:** Eduardo Nolla Silva Pereira, Luciana Sacilotto, Gabrielle D’Arezzo Pessente, Cinthya Guirao, Mariana Lombardi Peres de Carvalho, Alexandre da Costa Pereira, Francisco Carlos da Costa Darrieux, Maurício Ibrahim Scanavacca

**Affiliations:** 1 Universidade de São Paulo Faculdade de Medicina Hospital das Clínicas São Paulo SP Brasil Universidade de São Paulo – Faculdade de Medicina Hospital das Clínicas – Instituto do Coração, São Paulo, SP – Brasil

**Keywords:** Recém-nascido, Síndrome do QT longo, Taquicardia Ventricular, Mexiletina/uso terapêutico, Torsades de Pointes, Reanimação Cardiopulmonar

## Introdução

A síndrome do QT longo tipo 3 (SQTL3) é uma canalopatia de alta letalidade. Está associada ao déficit de fechamento dos canais tardios de sódio, decorrentes de mutações no gene *SCN5A* , de padrão autossômico dominante, responsável por 7-10% de todas as síndromes do QT longo (SQTL).^1^ A apresentação inicial pode ter amplo espectro, desde assintomáticos, até morte súbita no primeiro ano de vida.^2^ A adição do bloqueador do canal de sódio classe IB (mexiletina) ao propranolol ou nadolol é considerada um tratamento gene-guiado, já que seu benefício está comprovado na SQTL3.^3^ Em alguns países, como o Brasil, é inviável tratar pacientes com SQTL3, pela indisponibilidade da mexiletina.

A seguir, apresentaremos um caso grave de uma criança com SQTL3, que evoluiu com múltiplas terapias pelo cardiodesfibrilador implantável (CDI), pela dificuldade de acesso à mexiletina no Brasil.

## Relato de caso

**Figura 1:**
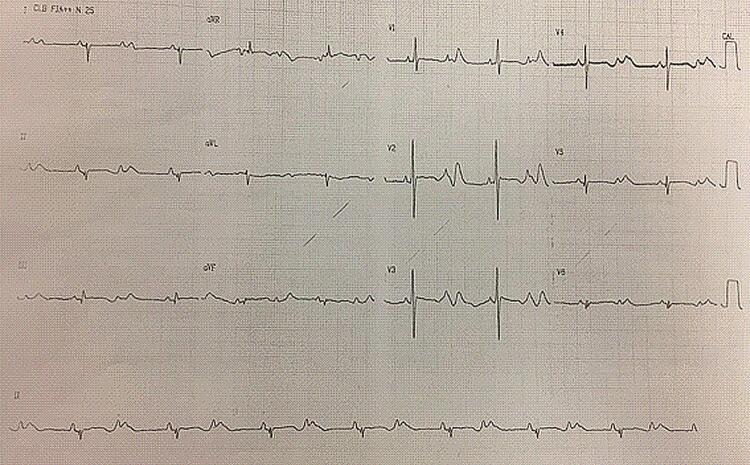
– Primeiro eletrocardiograma, realizado um dia após o nascimento da paciente. Ritmo sinusal com bloqueio atrioventricular 2:1 e prolongamento do QT.

**Figura 2:**
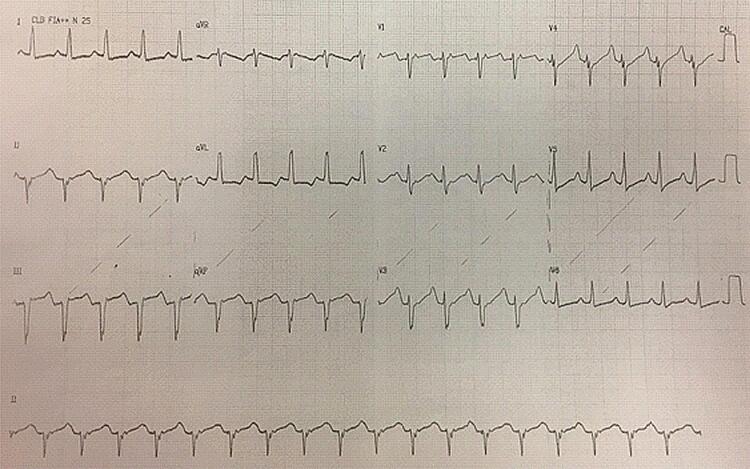
– Eletrocardiograma mostrando ritmo ventricular estimulado pelo marca-passo e prolongamento do QT.

Trata-se de uma criança, do sexo feminino, filha de pais saudáveis, sem consanguinidade. Nasceu de parto cesáreo, devido a arritmia intrauterina (taquicardia alternada com bradicardia). Quando recém-nascida, apresentou múltiplos episódios de taquicardia ventricular não sustentada polimórfica (TVNSp). O eletrocardiograma (ECG) basal mostrava bloqueio atrioventricular (BAV) 2:1 e intervalo QT prolongado ( Figura 1 ). Foi iniciado tratamento com propranolol 1mg/kg/dia, e, devido à piora da bradicardia, optou-se pelo implante de marca-passo endovenoso unicameral ( Figura 2 ).

Aos três meses de idade, a paciente apresentou fibrilação ventricular, sendo prontamente reanimada, com retorno à circulação espontânea. Pela suspeita de SQTL,^3^ mesmo na ausência do teste genético para guiar tratamento e na indisponibilidade de mexiletina para teste terapêutico, optou-se por aumentar a dose de propranolol para 4,5mg/kg e acrescentar fenitoína. Houve melhora transitória da recorrência de TVNSp, e decidiu-se realizar simpatectomia cervicotorácica.

Aos sete meses, após novos episódios de cianose durante o sono, a criança foi levada ao hospital, onde apresentou três episódios de *torsades de pointes* (TdP), necessitando reanimação cardiopulmonar e desfibrilação. Nessa internação, implantou-se um CDI. Realizou-se genotipagem pela técnica de *next generation sequencing* (NGS), com um painel de 15 genes associado à síndrome do QT longo. Foi identificada uma variante *missense* no gene *SCN5A* , c.5287G>A, que determinou a troca de valina por metionina na posição 1763 (p.Val1763Met), localizada no domínio S6 transmembrana do canal de sódio. Essa variante é classificada como patogênica, de acordo com os critérios do American College of Medical Genetic and Genomic (ACMG). O rastreamento genético dos pais, por meio da técnica de Sanger, não revelou a variante do caso índice, confirmando uma variante *de novo* .

Aos 14 meses de idade, a paciente apresentou múltiplos choques do CDI, especialmente durante o sono, e a mexiletina ainda não havia sido comprada, pelo alto custo da medicação importada. Não foi iniciado tratamento com propafenona, devido ao conhecimento prévio de pacientes com a mesma mutação nos quais esse medicamento desmascarou o padrão de Brugada. A equipe assistencial importou o medicamento, sendo a dose atingida de 8mg/kg/dia, em associação ao propranolol. A criança apresentou melhora clínica importante, sem novos eventos arrítmicos. No momento, aos 3 anos de idade, a paciente experimentou recorrência de arritmia após 1 ano de falta de mexiletina, e possui o desenvolvimento neurológico apropriado.

## Discussão

Apresentamos o caso de uma criança com SQTL3, com manifestações arrítmicas graves e raras desde o nascimento. A bradicardia por BAV 2:1 e os episódios recorrentes de TdP neonatal são observados, com mais frequência, em pacientes com SQTL3, especialmente em mutações *de novo* no gene *SCN5A.* ^4^

A bradicardia fetal é um fenômeno conhecido em pacientes com síndrome do QT longo. A bradicardia sinusal é mais observada em pacientes com SQTL tipo 1.^5^ Considera-se que BAV 2:1 com SQTL resulte da relação entre a curta duração do ciclo sinoatrial e o período refratário ventricular muito longo dos pacientes com SQTL. A presença de BAV 2:1 é indicador de alto risco de arritmias potencialmente fatais, como observada em nossa paciente.^6^

Okuwakiet al.^7^ descreveram uma criança com fenótipo muito semelhante ao observado em nosso atendimento, inclusive portadora da mesma variante *de novo* Val1763Met, que apresentou controle do intervalo QT e das arritmias ventriculares com mexiletina endovenosa.^7^ Yao et al.^8^ descreveram um caso de SQTL com fenótipo arritmogênico grave, que apresentou melhora importante após propranolol e mexiletina empírica, sem diagnóstico molecular.^8^ Schulze-Bahr et al. descreveram um caso similar de síndrome do QT longo com BAV 2:1 e arritmias ventriculares.^6^

Os bloqueadores dos canais de sódio, incluindo flecainida, ranolazina e mexiletina, compartilham locais de ligação na região do poro interno do canal de sódio Nav1.5, e têm eficácia documentada em portadores de SQTL.^3^ A propafenona, único bloqueador dos canais de sódio disponível no Brasil, foi evitada devido ao risco de exacerbação do padrão de Brugada e pró-arritmias como fibrilação ventricular. Há diversos relatos na literatura alertando quanto ao risco de pró-arritmia em pacientes com SQTL3 e mutações nas regiões próximas ao resíduo 1763.^9^ A ranolazina, embora liberada para uso como antianginoso no Brasil, apresenta complexo perfil metabólico em crianças. Tan et al.,^10^ descreveram um relato de caso em criança, comprovando uma meia-vida muito curta e diversas interações medicamentosas nessa faixa etária, que poderiam gerar efeitos colaterais pró-arrítmicos significativos.^10^

Dessa maneira, a falta de escolhas alternativas e a indisponibilidade da mexiletina levou a um atraso importante e potencialmente fatal no tratamento do caso apresentado. Após introdução da mexiletina, houve uma redução importante no numero de terapias pelo CDI.

## Conclusão

Esse caso ilustra a complexidade e responsabilidade assumida pela equipe médica no tratamento dessa criança no Brasil, com manifestação precoce da doença. O difícil acesso à mexiletina, necessária para terapia gene-guiada em pacientes de alto risco com SQTL3 gerou importante repercussão em sua qualidade de vida e risco de morte súbita.
