# Supplementary material for: Mexiletina em um Recém-Nascido com Síndrome do QT Longo Tipo 3: Quando o Acesso se Impõe à Urgência
Source: Arq Bras Cardiol. 2022 May 4;118(5):989–91. [Article in Portuguese] doi: 10.36660/abc.20210533 (PMC9368877; doi:10.36660/abc.20210533)
Supplement: Translation

# Translation: Mexiletine in a Newborn with Type 3 Long QT Syndrome: When Access is Difficult

**Authors:** Eduardo Nolla Silva Pereira, Luciana Sacilotto, Gabrielle D’Arezzo Pessente, Cinthya Guirao, Mariana Lombardi Peres de Carvalho, Alexandre da Costa Pereira, Francisco Carlos da Costa Darrieux, Maurício Ibrahim Scanavacca

**Affiliations:** 1 Brasil Universidade de São Paulo – Faculdade de Medicina Hospital das Clínicas – Instituto do Coração, São Paulo, SP – Brasil

**Keywords:** Infant, Newborn, Long QT syndrome, Tachycardia, Ventricular, Mexiletine/therapeutic, Torsades de Pointes, Cardiopulmonary Resuscitation

## Introduction

Long QT syndrome type 3 (LQT3) is a highly lethal channelopathy. It is associated with delayed closing of sodium channels, resulting from mutations in the *SCN5A* gene, with an autosomal dominant pattern, responsible for 7-10% of all long QT syndromes (LQTSs).^1^ The initial presentation can have a wide spectrum, from asymptomatic to sudden death in the first year of life.^2^ The addition of class IB sodium channel blocker (mexiletine) to propranolol or nadolol is considered a gene-guided treatment, as its benefit is proven in LQT3.^3^ In some countries, such as Brazil, it is not feasible to treat patients with LQT3, because of the unavailability of mexiletine.

We present here a severe case of a child with LQT3, who underwent multiple implantable cardioverter-defibrillator (ICD) therapies because of difficult access to mexiletine in Brazil.

## Case Report

The patient was a girl of healthy parents without consanguinity. She was born by cesarean section because of intrauterine arrhythmia (tachycardia alternating with bradycardia). As a newborn, she had multiple episodes of polymorphic non-sustained ventricular tachycardia (pNSVT). Baseline electrocardiogram (ECG) showed a 2:1 atrioventricular block (AVB) and prolonged QT interval ( Figure 1 ). Treatment with propranolol 1 mg/kg/day was started. and because of worsening of bradycardia, we decided on implantation of a single-chamber intravenous pacemaker ( Figure 2 ).

**Figure 1:** – First electrocardiogram, performed one day after the patient’s birth. Sinus rhythm with 2:1 atrioventricular block and QT prolongation.

**Figure 2:** – Electrocardiogram showing pacemaker-stimulated ventricular rhythm and QT prolongation.

At three months of age, the patient developed ventricular fibrillation and was promptly resuscitated, with return to spontaneous circulation. Because of suspicion of LQT,^3^ even in the absence of genetic testing to guide treatment and the unavailability of mexiletine for therapeutic testing, we chose to increase the dose of propranolol to 4.5 mg/kg and add phenytoin. There was a transient improvement in the recurrence of pNSVT, and we decided to perform cervicothoracic sympathectomy.

At seven months, after new episodes of cyanosis during sleep, the child was taken to the hospital, where she had three episodes of torsades de pointes (TdP), requiring cardiopulmonary resuscitation and defibrillation. During this hospitalization, an ICD was implanted. Genotyping was performed using next-generation sequencing, with a panel of 15 genes associated with long QT syndrome. A missense variant was identified in the *SCN5A* gene, c.5287G>A, which determined the substitution of valine by methionine at position 1763 (p.Val1763Met), located in the S6 transmembrane domain of the sodium channel. This variant is classified as pathogenic according to the American College of Medical Genetics and Genomics (ACMG) criteria. Genetic screening of the parents, using the Sanger technique, did not reveal the index case variant, confirming a *de novo* variant.

At 14 months of age, the patient had multiple ICD shocks, especially during sleep, and mexiletine had not yet been purchased because of the high cost of imported drugs. Treatment with propafenone was not started, because of previous knowledge of patients with the same mutation in whom this drug unmasked the Brugada pattern. The care team imported the drug, and the dose reached 8 mg/kg/day, in combination with propranolol. The child showed substantial clinical improvement, without new arrhythmic events. At 3 years of age, the patient has experienced recurrence of arrhythmia after 1 year of lack of mexiletine and has appropriate neurological development.

## Discussion

We present the case of a child with LQT3, with severe and rare arrhythmic manifestations since birth. 2:1 AVB bradycardia and recurrent episodes of neonatal TdP are seen more frequently in patients with LQT3, especially in *de novo* mutations in the *SCN5A* gene.^4^

Fetal bradycardia is a known phenomenon in patients with LQTS. Sinus bradycardia is more commonly observed in patients with LQTS type 1.^5^ It is considered that 2:1 AVB with LQTS results from the relationship between the short duration of the sinoatrial cycle and the very long ventricular refractory period of patients with LQTS. The presence of 2:1 AVB is an indicator of a high risk of potentially fatal arrhythmias, as observed in our patient.^6^

Okuwaki et al.^7^ described a child with a phenotype very similar to that observed in our care, including a carrier of the same *de novo* Val1763Met variant, who showed control of the QT interval and ventricular arrhythmias with intravenous mexiletine.^7^ Yao et al.^8^ described a case of LQTS with severe arrhythmogenic phenotype, which showed significant improvement after empirical propranolol and mexiletine, without molecular diagnosis.^8^ Schulze-Bahr et al. described a similar case of LQTS with 2:1 AVB and ventricular arrhythmias.^6^

Sodium channel blockers, including flecainide, ranolazine and mexiletine, share binding sites in the inner pore region of the Nav1.5 sodium channel, and have documented efficacy in LQTS carriers.^3^ Propafenone, the only sodium channel blocker available in Brazil, was avoided because of the risk of exacerbation of the Brugada pattern and ventricular fibrillation as proarrhythmias. There are several reports in the literature warning about the risk of proarrhythmia in patients with LQTS3 and mutations in the regions close to residue 1763.^9^ Ranolazine, although approved for use as an antianginal agent in Brazil, has a complex metabolic profile in children. Tan et al.^10^ described a case report in a child, demonstrating a very short half-life and several drug interactions in this age group, which could generate significant proarrhythmic side effects.^10^

Thus, the lack of alternative choices and the unavailability of mexiletine led to an important and potentially fatal delay in the treatment of the case presented. After the introduction of mexiletine, there was a significant reduction in the number of ICD therapies.

## Conclusion

This case illustrates the complexity and responsibility assumed by the medical team in the treatment of this child with early manifestation of the disease, in Brazil. Difficult access to mexiletine, which is necessary for gene-guided therapy in high-risk patients with LQT3, has had a critical impact on their quality of life and risk of sudden death.
